# The Pathogenesis of Endometriosis: Molecular and Cell Biology Insights

**DOI:** 10.3390/ijms20225615

**Published:** 2019-11-10

**Authors:** Antonio Simone Laganà, Simone Garzon, Martin Götte, Paola Viganò, Massimo Franchi, Fabio Ghezzi, Dan C. Martin

**Affiliations:** 1 Department of Obstetrics and Gynecology, “Filippo Del Ponte” Hospital, University of Insubria, Piazza Biroldi 1, 21100 Varese, Italy; simone.garzon@univr.it (S.G.); fabio.ghezzi@uninsubria.it (F.G.); 2 Department of Gynecology and Obstetrics, Münster University Hospital, D-48149 Münster, Germany; mgotte@uni-muenster.de; 3 Reproductive Sciences Laboratory, Division of Genetics and Cell Biology, San Raffaele Scientific Institute, Via Olgettina 60, 20136 Milan, Italy; vigano.paola@hsr.it; 4 Department of Obstetrics and Gynecology, AOUI Verona, University of Verona, Piazzale Aristide Stefani 1, 37126 Verona, Italy; massimo.franchi@univr.it; 5 School of Medicine, University of Tennessee Health Science Center, 910 Madison Ave, Memphis, TN 38163, USA; danmartin46@gmail.com; 6 Virginia Commonwealth University, 907 Floyd Ave, Richmond, VA 23284, USA

**Keywords:** endometriosis, pathogenesis, genetics, epigenetics, immunology

## Abstract

The etiopathogenesis of endometriosis is a multifactorial process resulting in a heterogeneous disease. Considering that endometriosis etiology and pathogenesis are still far from being fully elucidated, the current review aims to offer a comprehensive summary of the available evidence. We performed a narrative review synthesizing the findings of the English literature retrieved from computerized databases from inception to June 2019, using the Medical Subject Headings (MeSH) unique ID term “Endometriosis” (ID:D004715) with “Etiology” (ID:Q000209), “Immunology” (ID:Q000276), “Genetics” (ID:D005823) and “Epigenesis, Genetic” (ID:D044127). Endometriosis may origin from Müllerian or non-Müllerian stem cells including those from the endometrial basal layer, Müllerian remnants, bone marrow, or the peritoneum. The innate ability of endometrial stem cells to regenerate cyclically seems to play a key role, as well as the dysregulated hormonal pathways. The presence of such cells in the peritoneal cavity and what leads to the development of endometriosis is a complex process with a large number of interconnected factors, potentially both inherited and acquired. Genetic predisposition is complex and related to the combined action of several genes with limited influence. The epigenetic mechanisms control many of the processes involved in the immunologic, immunohistochemical, histological, and biological aberrations that characterize the eutopic and ectopic endometrium in affected patients. However, what triggers such alterations is not clear and may be both genetically and epigenetically inherited, or it may be acquired by the particular combination of several elements such as the persistent peritoneal menstrual reflux as well as exogenous factors. The heterogeneity of endometriosis and the different contexts in which it develops suggest that a single etiopathogenetic model is not sufficient to explain its complex pathobiology.

## 1. Introduction

The etiopathogenesis of endometriosis is a multifactorial process resulting in a heterogeneous disease [1]. Its origin may be from Müllerian or non-Müllerian stem cells. These could include stem cells of the endometrial basal layer, Müllerian remnants, bone marrow, or the peritoneum. Furthermore, the innate ability of endometrial stem cells to regenerate cyclically under the influence of estrogen followed by estrogen/progesterone stimulation and then hormonal withdrawal seems to play a key role. The presence of such cells in the peritoneal cavity and what leads to the development of endometriosis is a complex process with a large number of interconnected factors potentially both inherited and acquired [2]. Genetic studies have confirmed a complex genetic nature [3]. At the same time, the epigenetic mechanisms underlying endometriosis support the processes that promote the acquisition and maintenance of immunologic, immunohistochemical, histological, and biological aberrations that characterize both the eutopic and ectopic endometrium in patients affected by endometriosis. This may be related to the particular combination of factors linked menstrual reflux into the peritoneal cavity as well as exogenous factors [4]. Once started, the process is variable and can lead to the development of endometriosis or can reach a limit to its growth and then stabilize or regress. As a result, the heterogeneity of endometriosis and the different phenotypes suggest that a single etiopathogenetic explaining model is not sufficient.

### What is Endometriosis?

Endometriosis is a common, benign, inflammatory, generally gynecologic disease that includes the presence and growth of dysfunctional endometrial-like glands and stroma often with reactive fibrosis and muscular metaplasia outside the uterus [5]. It is associated with pelvic pain and subfertility in reproductive age women and can severely compromise the quality of life of affected women [6,7,8,9,10,11] and require extensive surgery when more conservative treatment options fail [12,13]. The prevalence rate of symptomatic endometriosis is estimated to be 10% with an incidence of about 2-7/1000 women per year and a further 11% of undiagnosed cases, although there are only a few studies with well-estimated prevalence and incidence of endometriosis in the general population [14,15,16] and some suggesting that many, if not all, women have endometriosis as a transient phenomenon [17,18].

Since the introduction of the term “endometriosis” and its pathogenesis theories by Sampson [19,20,21], extensive basic and clinical research concerning the etiopathogenesis of endometriosis has been carried out. However, the exact origin and mechanism of endometriosis development remain theoretical. The growing body of evidence confirms the multifactorial nature of endometriosis that is the result of the combined contribution of anatomical, hormonal, immunological, reactive, estrogenic, genetic, epigenetic, and environmental factors in affected women [7]. These multiple interconnected factors may explain the complex and heterogeneous presentations of the disease with different locations, appearances, developments, and hormone responsiveness. The heterogeneity and differences among the three main classes of endometriosis presentation (peritoneal, ovarian, and deep infiltrating endometriosis) are such as to suggest different pathogenetic pathways [22,23]. Moreover, a generally accepted hypothesis is that endometriosis is a phenomenon that may occur intermittently in all women during menstrual cycles, but that develops in a disabling disease only in a subset of women [17,18,24,25]. Understanding the multiple pathogenetic pathways underlining the development of endometriosis is of paramount importance, as they may have implications in the prevention, diagnosis, treatment, and prognosis of the disease [6].

## 2. Theories on the Origin of Endometriosis

The first question about the pathogenesis of endometriosis was about the origin of the endometrial-like glands and stroma that constitute the disease. Several hypotheses have been proposed since 1870 [26], some of only historical interest and some that are now considered the most plausible; nevertheless, none is able to completely explain the pathogenesis of endometriosis and all the different presentations of the disease [27]. Overall, all the proposed hypotheses for the cell origin can be categorized into two main theories: the in-situ theory and the transplantation theory.

### 2.1. The in Situ Theory

All the hypotheses belonging to this category are based on the concept that stroma and glands of endometrial-like tissue of endometriosis originate in-situ from the local tissues by metaplasia or by embryological origin. This hypothesis was proposed by Waldeyer in 1870 [26], who suggested that endometriosis develops from the germinal epithelium of the ovary by “metaplasia”. Later, Von Recklinghausen in 1895 and Russell in 1899 introduced the concept of embryological origin from mesonephric/Wolffian remnants and Müllerian remnants, respectively [27]. 

The terms metaplasia and differentiation, whether these refer to the endometrium, to endometriotic cells, or pluripotent stem cells to endometriotic cells [28], are both used in this manuscript based on the original source document. We do not attempt to distinguish the differences between those two terms with overlapping meanings.

The Müllerian remnants hypothesis explains endometrial-like tissue as having developed from differentiation and proliferation of embryonic cell rests that are constituted by misplaced cells of primitive endometrial tissue along the migratory pathway of Müllerian ducts [29,30]. The cells spread across the posterior pelvic floor due to aberrant migration and differentiation during organogenesis of the female genital tract. Based on this hypothesis, “Müllerianosis” and “Secondary Müllerian system” theories were proposed as theoretical explanations of cell origin and dissemination of Müllerian-type epithelium outside the expected area of Müllerian duct development, including endometriosis, adenomyosis, endosalpingiosis, and endocervicosis [29,30]. However, embryological studies support the presence of Müllerian rests near the normal deep cul-de-sac area and not in other sites such as the ovary, sigmoid colon, appendix, or more distal sites such as the diaphragm and pleura [31]. 

The “Müllerianosis” hypothesis might explain that endometriosis is often found in the cul-de-sac, uterosacral ligaments, and medial broad ligaments; that peritoneal pockets with and without endometriosis have been associated with congenital tract malformations; and that endometriosis seems to have higher prevalence even in women with non-obstructive Müllerian abnormalities [32]. Moreover, this hypothesis is supported by the fact that is able to explain the presence of endometriosis in women with Mayer–Rokitansky–Küster–Hauser syndrome, in adolescents before or shortly after menarche , and in human female fetuses; where organoid structures outside uterine cavity resembling primitive endometrium were reported [31,33,34,35]. As previously noted, Signorile et al. [31] found CD10 positive remnants in the deep cul-de-sac only. Finally, Müllerian remnants or coelomic metaplasia in prostate and utricle may explain rare cases of endometriosis reported in males, after long-term high doses of estrogens for prostate carcinoma [36,37]. Immunohistochemical studies in men support both Müllerianosis and metaplasia theories [37].

Coelomic metaplasia or the stem cell differentiation hypothesis is based on the fact that, in the embryonic phase, the coelomic epithelium gives rise to both mesothelium of serosae and the epithelium lining of the cavity of Müllerian ducts, which forms the endometrium in the uterine body. This hypothesis explains endometriosis as developing from the metaplastic transformation of germinal ovarian epithelium and/or peritoneum serosa [38,39]. These metaplastic changes are supposed to occur secondary to hormonal influences [40,41], inflammatory processes [42,43], or the action of one or several endogenous biochemical or immunological factors derived from eutopic endometrium, based on the “induction” theory [44,45]. The induction theory is based on animal model studies suggesting that specific cell-free endometrial products were capable of inducing the metaplasia of undifferentiated mesenchyme into endometrial epithelium and glands, although no endometrial stroma was found. On that basis, it was supposed that these substances, released by uterine endometrium, may diffuse into the lymphatic and bloodstream and induce the formation of endometriosis in distant parts of the body [44,45]. The coelomic metaplasia hypothesis and the Müllerian remnants hypothesis may explain endometriosis in the absence of menstruation; and, additionally, it may explain the presence of endometriosis outside the pelvis such as in the chest, diaphragm, pleura, and lungs [36,46], although direct infiltration through the diaphragm or dissemination through diaphragmatic fenestrations or perforations are retrograde possibilities.

The main strength of in-situ theories is that they are able to explain endometriosis in women without menses or endometrium. Nevertheless, several factors are against in-situ theories. If peritoneal cells can easily undergo metaplastic transformation, endometriosis should be observed more frequently in men, in the thoracic cavity, and with a uniform distribution in the peritoneum. Moreover, if coelomic metaplasia resembles common metaplasia, the incidence of endometriosis should increase with advancing age. Finally, although some evidence suggests that endometriosis has a higher prevalence even in women with non-obstructive Müllerian abnormalities [32], other studies reported endometriosis to be more frequent in patients with Müllerian anomalies and outflow obstruction, and not in Müllerian anomalies as a whole [47,48]. 

### 2.2. The Transplantation Theory

In this category, the hypotheses are based on the concept that the stroma and glands of endometriosis originate from the eutopic endometrium. Endometriosis is proposed as benign metastasis of eutopic endometrium, which is displaced from the uterine cavity to another location inside the body through different routes. Hematogenous, lymphatic, and iatrogenic (mechanical) spread of endometrial or endometriotic cells can explain all uncommon extraperitoneal locations [49]. However, the most popular theory was introduced by Sampson in 1927 based on clinical and anatomical observations. Sampson proposed the retrograde menstruation theory that concludes that most endometriosis derives from the reflux of eutopic endometrial fragments through the fallopian tubes during menstruation, with subsequent implantation, transition from endometrium to endometriosis, and growth on and into the peritoneum and the ovary [19,20,21]. In addition, Sampson recognized that retrograde menstruation could not explain all forms of endometriosis and suggested venous dissemination or metaplasia as alternate theories [19,20].

Over the years, a growing body of evidence has supported the “retrograde menstruation” theory, and it is now the most accepted hypothesis for most forms of endometriosis. The most important step was the demonstration that the tubal reflux of menstrual tissue is a common event in women with patent fallopian tubes documented in 76-90% of women [6]. Blood was found in peritoneal fluid by laparoscopy in 90% of women with patent tubes and only in 15% with occluded tubes during the peri-menstrual period [50], and endometrial epithelial cells have been isolated in the peritoneal fluid of women during the early proliferative phase [51]. The second step was the identification of viable endometrium, single cells, and glandular structures in the shed menstrual tissue [52]. The demonstration that reflux of viable endometrium in the peritoneal cavity is a common event in fertile age women was essential to consider the “retrograde menstruation” theory plausible.

Moreover, the anatomical distribution of endometriosis in the pelvis with higher prevalence in the left side than in the right side, that is compatible with anatomical differences between the right and left hemipelvis [53], and the distribution in the abdomen with higher prevalence in the right diaphragm than in the left following the counter-clockwise distribution of peritoneal fluid [54], further supports the “retrograde menstruation” theory [53,54,55]. 

However, the transplantation theories are unable to explain endometriosis in women with Mayer–Rokitansky–Küster–Hauser syndrome, in adolescents before or shortly after menarche, and in males [19,31,33,34,35,37]. Moreover, available evidence suggests that endometriosis is not simply a transplanted normal endometrium. Numerous differences in hormone receptor levels, as well as histological, morphological, and biological characteristics, were reported when comparing endometriosis with eutopic endometrium, with only limited similarities. Although Sampson recognized that endometriosis was different from endometrium "both in structure and in function” and noted a transition from one to the other [19,20], his 1920s observations of a transition do not include all of the inflammatory, chemical, immunologic, epigenetic and genetic changes that have been discovered the last 40 years. Those changes require additional understanding of the transformation of any Müllerian (endometrial or rest) or non-Müllerian cell to endometriosis. Furthermore, eutopic endometrium of affected women is reported to have similar alterations of endometriotic lesions, that are not found in the eutopic endometrium of healthy women. This supports the hypothesis that the primary defect might be rooted in eutopic endometrium of women with endometriosis, although the gap between the incidence of refluxed menstruation and the incidence of endometriosis highlights the presence of further mechanisms [2].

## 3. Behind the Origins of Endometriosis

Although the origin of endometrial-like tissue that constitutes endometriosis is theoretical and debated, it is generally agreed that endometriosis is a phenomenon that may occur in all women during reproductive age, close laparoscopic examination of otherwise healthy peritoneum and microscopic examination of resected bowel can reveal almost microscopic or minimal peritoneal lesions [56,57,58], and many, if not most, of the small lesions tend to resolve or become inactive spontaneously. Therefore, only a subset of women with endometriosis develops a disabling disease regardless of the tissue origin [17,18,24,25]. To understand the origin of endometriosis and the mechanisms that explain the development of endometriosis as a disabling disease instead of spontaneous resolution, in vitro and in vivo studies and immunohistochemical, genetic, and epigenetic analysis were conducted [59,60,61]. Some studies, typically based on in vitro and in vivo models, were developed to investigate the stepwise formation of endometriotic lesions in order to test different specific hypotheses of the origin of endometriosis and to identify mechanisms that allow endometriotic lesion development [59,60]. Conversely, other studies, typically immunohistochemical, genetic, and epigenetic analysis, focused the investigation on the identification of prerequisites for the development of the disease instead of spontaneous resolution, comparing ectopic with eutopic endometrium in affected women or comparing parameters between affected and unaffected women [54,62,63,64]. Overall, this growing body of evidence suggests that endometriosis is not simply ectopic endometrium, with many reported differences between the endometrial-like tissue of endometriosis and eutopic endometrium in affected women [65]. However, this data has not clarified when replanted endometrium or Müllerian remnants begin the transition from ectopic Müllerian tissue to endometriosis, or when non-Müllerian stem cells are committed to differentiate into endometriosis. At the same time, when women with symptomatic endometriosis are compared with women without or minimal disease, the differences were reported not only at the level of endometriosis implants but even at the level of eutopic endometrium [66], uterus [67], and peritoneal environment [68].

### 3.1. Comprehensive Models on the Origin of Endometriosis

The induction theory was tested by in vivo studies on rabbit models. Endometrium was implanted in the abdominal cavity, and tissue was histologically evaluated for seven days [44]. Endometrial implants degenerated during the first four days, and cysts and endometrium-like differentiation were observed in the next three days in the surrounding connective tissue. If the tissue was dissociated before implantation, better results were obtained. These findings were further confirmed in a study in which viable and ischemic endometrial tissue was implanted intraperitoneally in rabbits within Millipore filters, that allowed only chemical substances to pass due to the small pore size [45]. Endometrium-like epithelium and glands were observed in the connective tissue adjacent to the implants. Although these changes did not include stroma, these observations support the hypothesis that endometrial tissue liberates specific substances inducing undifferentiated mesenchyme to develop into endometrial tissue.

Conversely, investigating the possibility that retrograde menstruation of shed endometrium is the origin of endometriosis, a chicken chorioallantoic membrane (CAM) model, an in vitro model that uses the membrane covering the chicken embryo, was used to study the stepwise endometriotic lesion formation involved in this process [69]. Tissue can be transplanted onto CAM and interventions can be carried out, allowing the behavior of the tissue and the consequences of interventions to be observed. In order to visualize the different steps of “retrograde menstruation” theory, human menstrual endometrial fragments were collected and were transplanted onto the CAM. After 24, 48, and 72 hours, cross-sections of the CAMs were cut and immunohistochemically stained. After 24 hours, direct contact was present between menstrual tissue fragments and the CAM mesenchyme. After 48 h, the menstrual endometrium was reorganized inside the CAM mesenchyme, and after 72 hours, a complete endometrium with glands and stroma was present in the CAM mesenchyme. Moreover, blood vessels attracted from the CAM were present inside the endometrium fragment. This model showed that viable endometrium is necessary to form an endometriotic lesion, and that stroma and glands of shed menstrual endometrium are able to adhere to and degrade the matrix and to induce neo-angiogenesis in order to survive [70].

### 3.2. Role of Hormones

Why a transplanted or congenital ectopic endometrium develops into endometriosis is the source of much research. The causes of this development include research on the role of estrogen and estrogen receptors (ERs), the estrogen-dependent physiologic and molecular changes [71], the local levels of estrogen [71,72], the role of estrogen in macrophage-nerve interaction [72], the effects of environmental toxicants on estrogen signaling [73], and the intracellular estrogen production related to aromatase activity. In addition, the normal control of cyclic estrogen and progesterone requires activation and crosstalk of cAMP and progesterone mediated signaling pathways [74].

Intracellular production of estrogens has a key role in the pathogenesis of endometriosis, particularly in post-menopausal women [75], as well as of other benign and malignant diseases of the female reproductive tract. Aromatase P450 catalyzes the conversion of androgens to estrogens and is physiologically expressed in different human tissues, including ovaries and adipose tissue, but usually not in the endometrium [76,77]. 

In women with endometriosis, this enzyme has been found in both endometriotic tissue and eutopic endometrium [77,78]. Moreover, in endometriosis the protective action of 17β-hydroxysteroid dehydrogenase (17β-HSD) type 2 is lost due to enzymatic deficiency. 17β-HSD lowers the level of the strong 17β-estradiol, converting it into the weak estrone, modulating the exposure to estrogens action [79]. The local production of estrogens and the loss of protective mechanisms determine a higher estradiol level that characterizes both endometriosis and eutopic endometrium of affected women, as demonstrated by the higher estradiol level of menstrual effluent in women with endometriosis as compared to controls [80]. Moreover, the increased estrogen production in endometriotic lesions and eutopic endometrium determines a positive feedback loop resulting in further estrogen production through the induction of cyclo-oxygenase type 2 (COX-2) enzyme. The subsequently elevated levels of prostaglandin E2 further stimulates the aromatase activity [78,81].

Of interest, the local production of estrogen was reported as a result of the activation of tissue injury and repair (TIAR) mechanisms induced by microtrauma at the level of basal endometrial layer. The basal endometrial layer has stem cell characteristics and exhibits the potential for dislocation and proliferation, that was reported enhanced in women with endometriosis [82,83]. The fragments of basal endometrium dislocated into the peritoneal cavity may induce chronic inflammation and TIAR mechanisms, that activate local production of estrogen, proliferation, and infiltrative growth resulting in endometriosis [67,84].

**Figure 1:**
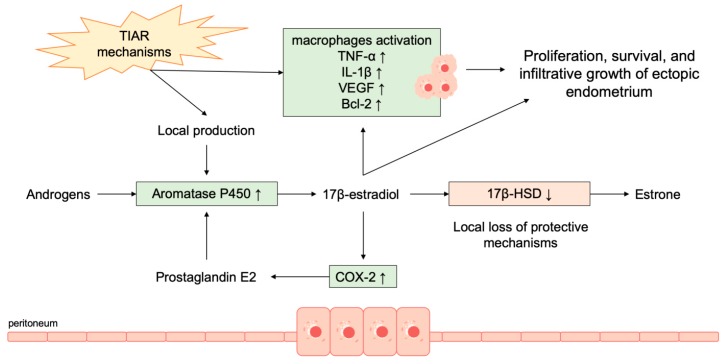
Summary of the mechanisms underlining the key role of estrogens in the pathogenesis of endometriosis. Tissue injury and repair (TIAR); Tumor necrosis factor (TNF); Interleukins (IL); Vascular endothelial growth factor (VEGF); Antiapoptotic protein B cell lymphoma 2 (Bcl-2); 17β-hydroxysteroid dehydrogenase (17β-HSD).

The estrogenic microenvironment was reported able to activate macrophages into peritoneum with the consequent secretion of pro-inflammatory cytokines such as tumor necrosis factor-α (TNF-α) and interleukin-1β (IL-1β) that stimulate the activation of NFkB. Moreover, these mechanisms induce vascular endothelial growth factor (VEGF) expression, cell cycle activation, and activation of the anti-apoptotic gene Bcl-2 [7,85] (Figure 1).

The key role of estrogens in endometriotic tissue survival and development is mediated by ERs. Endometriotic tissue development was reported suppressed by ER-selective modulators inhibiting estrogen receptor alfa (ER*α*) or beta (ER*β*) [86], as well as ectopic implants did not develop normally in ER*α*- or ER*β*-knockout mice [87,88]. ERs have similar affinity for estrogens and are transcriptional factors for similar subset of genes. Nevertheless, the differences between target genes, estrogen affinity, and tissue distribution of ER*α* and ER*β* homodimers as well as ER*α*/ER*β* heterodimer explain the reciprocal inhibitory and regulatory functions, as well as the different roles [89]. Although ER*α* was historical investigated due to his higher prevalence in the uterus and the supposed inhibitory effect of ER*β* in the eutopic endometrium [90], in the endometriotic tissue ER*α* was reported having a normal expression level as compared to normal endometrium. Conversely, ER*β* was reported overexpressed, determining an inversion of ER*β* to ER*α* ratio as compared to eutopic endometrium [91]. On that basis, it was supposed that both the high estrogens concentration and the overexpression of ER*β* are involved in the estrogen-based ectopic tissue survival and development. At the cytoplasmatic level, ER*β* was reported involved in the inhibition and disruption of TNF-*α*-induced apoptosis signaling [88]. At nuclear level, ER*β* was identified involved in the direct activation of the NFkB pathway and the radical oxygen species detoxification system, that are able to improve cell survival and cell escaping from immune clearance [92]. At the same time, ER*β* was related to the upregulation of hypoxia-induced signaling, epithelial mesenchymal transition signaling, and cytoskeleton components, that are all involved in the invasion and progression of endometriotic implants [92].

The synergistic counterpart of estrogen overproduction and ERs overexpression is the progesterone resistance in endometriotic tissue, that impedes to modulate genes involved in the decidualization, cell cycle regulation, and estrogen response inhibition [93]. The progesterone resistance is a characteristic of the endometriotic tissue as compared to the eutopic endometrium, although it was identified in the eutopic endometrium of affected women as compared to controls [94].

The main mechanism involved in the progesterone resistance is the downregulation of progesterone receptor (PR) in the ectopic tissue, that determines a variation in the expression of progesterone target genes, such as the gene coding the 17β-HSD [93,95].

The pathways potentially underlining the PR suppression are multiple. The concentration of pro-inflammatory cytokines, such as TNF-α and IL-1β involved in the chronic inflammation and TIAR mechanisms, is reported directly correlated with PR expression [96]. The activation of NFkB pathway by inflammation signaling determines a direct interaction with PR thorough an antagonist effect [97]. Similarly, the persistent phosphorylation of AKT determined by inflammation is involved in the inhibition of PR expression [98].

These mechanisms explain the progesterone resistance as an acquired characteristic of the endometriotic tissue versus an individual predisposition. This is further supported by the inconsistent results provided by genetic studies [99] and the involvement of epigenetic mechanisms, such as the methylation of the gene and related promoter coding for the PR [100], and the higher expression of miRNAs blocking the estrogen-dependent PR expression [101].

### 3.3. The Peritoneal Microenvironment and the Role of Immune Surveillance

The peritoneal fluid is produced by peritoneal and, mainly, ovarian exudation. It is a microenvironment that contains different cells, such as immune cells, endometrial cells, and red blood cells, which produce and secrete growth factors, angiogenic factors, and cytokines, that are able to affect processes in the abdominal cavity [102]. Of note, studies reported shed endometrial cells differing from eutopic cells; this may be explained by the different environments of bloodstream as compared to the peritoneal fluid [2]. In the abdominal cavity, the menstrual effluent determines an inflammatory response, of which physiological role is to clear the ectopic cells and tissue. Neutrophils, phagocytic leukocytes, and chemotactic leukocytes are attracted from the circulation, where an increased influx of bone marrow-derived cells is physiologically observed before the menstruation onset. Approximately 70-80% are macrophages CD14+, 20% are natural killer cells (NK cells) CD56+, and 10% are T-cells CD3+. This system is suggested to be overwhelmed or insufficient in women with endometriosis [103,104,105]. Shorter intervals and longer menstrual periods with heavy blood flow, that are often reported in women with endometriosis, may result in larger amounts of endometrial tissue collected in the abdominal cavity that overwhelms this system of cleansing [106,107]. Larger endometrial tissue fragments may provide protection from enzymatic and phagocytic activity to cells residing inside, that continue to produce angiogenic factors due to continued hypoxia. Moreover, the overwhelmed capacity to clean the peritoneum due to excessive refluxed endometrium may explain the higher prevalence of some anatomical defects reported in women with endometriosis, such as uterine malformations that prevent or disturb normal antegrade menstruation or that determine dysfunctional retrograde contractions [47,48,108,109]. 

Moreover, in women with endometriosis, endometrial cells were more resistant to the cytolytic action of autologous peritoneal macrophages than in healthy controls [110]. The cytotoxicity of NK cells against endometrial cells was reported as decreased with an inverse correlation with the stage of the disease [111]. Protection against the cytotoxicity of peritoneal NK cells seems to be provided by an altered antigenicity due to the overexpression of human leukocyte antigen class I [62,112]. Additionally, the eutopic endometrium of women with endometriosis releases higher levels of the soluble form of intercellular adhesion molecule-1 (sICAM-1) than those of women without endometriosis; and the ectopic endometrial cells express higher levels of sICAM-1 when compared to their eutopic endometrium. sICAM-1 modulates the cytotoxic activity of NK and CD8+ cells competing with ICAM-1 to bind leukocyte function antigen-1 (LFA-1). Binding of sICAM-1 to LFA-1 impedes leukocytes to bind ICAM-1 on the surface of target cells, preventing leukocyte activation [113,114,115]. The inflammatory response in endometriosis is further accentuated by the increased expression and activity of COX- 2, interleukins, and oxidative stress that act through the mitogen-activated protein kinase (MAPK) pathways. The subsequent dysregulation of MAPK signaling pathways increases inflammation, thereby recruiting immune cells and amplifying the inflammatory response. Moreover, MAPK signaling increases expression of growth factors, determines the development of pain and hypersensitivity to pain, and induces antiapoptotic signals. Of note, the dysregulation of apoptotic pathways and subsequent resistance to apoptosis contribute to the failure of immune clearance [116,117]. 

The failure to remove fragments of menstrual effluent from the abdominal cavity induces excessive local inflammation with further and persistent activation of macrophages, which may secrete an altered pattern of cytokines and chemokines. Evidence suggests that the number and activity level of peritoneal macrophages are higher in women with endometriosis, but their cytotoxic power is reduced [118,119,120]. Compared to physiology, macrophages in the peritoneum of affected women are not destroyed after completing their functions due to overexpression of the antiapoptotic protein Bcl-2, which protects them from apoptosis [121]. Peritoneal macrophages imbalance in M1 and M2 macrophages was reported in both eutopic and ectopic endometrium with upregulation of M2 type as compared to M1 type. Compared to M1 macrophages, which produce inflammatory cytokines and eliminate microorganisms and defective cells, the M2 macrophages modulate adaptive immune response, scavenge cellular debris, induce tissue repair, and induce angiogenesis. On that basis and experiments with macrophage depletion, M2 macrophages are supposed to have a key role in endometriosis development [116,122]. Moreover, smaller amounts of pro-inflammatory cytokines modulating the activation of macrophages (IL-6, IL-13, and IL-10 family) were produced and liberated in the eutopic endometrium of women with endometriosis as compared to the endometrium of healthy controls, reducing their cytotoxic capacity in the endometrium [120,123]. Production of IL-6 and MAPK activation in endometriotic cells are furthermore regulated by the proteoglycan Syndecan-1 (CD138), which also acts as a modulator of leukocyte and dendritic cell recruitment in mouse models of inflammation [124,125,126]. Studies reported that pro-inflammatory cytokines of Th1 profile are prevalent in the early stages, while these change to a Th2 profile in late stages, exerting an immunosuppressive effect and activating tissue injury-repair mechanisms [127]. This is consistent with data reporting reduced activity of cytotoxic T cells, a relative reduction of Th1 cell numbers, and a higher CD4/CD8 ratio in women affected by endometriosis as compared to healthy controls. Moreover, women with endometriosis were reported with both increased number and activation of B cells with an associated higher production of antibodies and higher numbers of regulatory T cells [116,128,129]. It is supposed that this altered inflammatory response may favor survival and implantation of ectopic endometrium with extracellular matrix (ECM) remodeling and angiogenesis as well as may cause metaplasia of the peritoneum or the development of Müllerian remnants, particularly overexpression of IL-1, IL-8, TNF-*α* [6,7]. Of interest, it is still unclear whether this altered peritoneal microenvironment is a cause or a consequence of endometriosis. Moreover, the presence of this altered inflammatory microenvironment could favor the implantation and development of endometriosis from refluxed endometrium or induce coelomic metaplasia of in situ mesothelium [20].

### 3.4. Apoptosis Defects

Cell turnover in human endometrium is regulated by apoptosis, which eliminates senescent cells from the functional layer during menses. Although apoptosis is regulated by several genes with variable expression during the menstrual cycle (bax, c-myc, and P53 induce it, while sentrin, B-cell lymphoma/leukemia-xL, and Bcl-2 inhibit it), the variation of endometrial apoptosis in the menstrual cycle seems to be primarily modulated by ovarian steroids through the up- and downregulation of Bcl-2 and bax expression, from the expression level of bax depends on the Bcl-2 action. Bcl-2 maximum expression was reported during the proliferative phase when the estrogens production and the expression of receptors in glandular cells is greatest [85,130,131,132].

**Figure 2:**
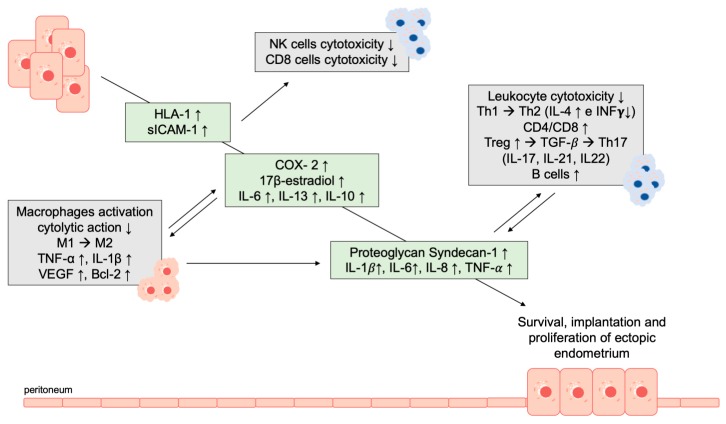
Immunoescaping mechanisms involved in the etiopathogenesis of endometriosis. Human leukocyte antigen class I (HLA-1); Intercellular adhesion molecule-1 (sICAM-1); Natural killer (NK); Cyclo-oxygenase type 2 (COX-2); Interleukins (IL); Tumor necrosis factor (TNF); Vascular endothelial growth factor (VEGF); Antiapoptotic protein B cell lymphoma 2 (Bcl-2); Interferon (INF); transforming growth factor-β (TGF-β).

In women with endometriosis, the eutopic endometrium is reported to exhibit significantly reduced apoptosis compared to women without endometriosis, particularly in the late secretory, menstrual, and early proliferative cycle phases. This may explain a reduced percentage of apoptotic cells and a greater number of surviving cells entering the peritoneal cavity, which is a prerequisite for the development of endometriosis [85,133,134,135]. The resistance to apoptosis could be related to different mechanisms. Inappropriate signal transduction was related to the dysregulated expression of proteins involved in the modulation of apoptosis, such as the increased expression of Bcl-2, that along with bax represent the key proteins of apoptosis regulation in endometriosis [85]. Compared to the cyclical activity of mTOR in eutopic endometrium, mTOR in endometriosis is constantly activated with persistent inhibition of cell autophagy and apoptosis [136]. Further studies identified the altered and excessive expression of the soluble-FasL in women with endometriosis, with subsequent dysregulated interaction between Fas and FasL that represent a possible cause of apoptosis resistance in endometriotic cells, in addition to immunoescaping. Of note, concomitant induced expression of FasL in stromal cells seems to mediate apoptosis of activated immune cells [116,137]. A constant source of TNF-α that initiates and modulates apoptosis during menses, and the absence of apoptosis induced by signals from adhesion receptors in cells that do not adhere to the peritoneal mesothelium, such as the E-cadherin suppression, are further mechanisms reported related to escape from apoptosis in endometriosis [131,135]. Of note, accumulating evidence suggests that apoptotic resistance has a key role in the “immunoescaping” of endometriotic cells from immune homeostasis of the peritoneal microenvironment [136] (Figure 2).

### 3.5. Cell-Matrix and Cell-Cell Adhesion

Dysregulated cell-cell and cell-matrix adhesion has a role in the development of endometriosis [138]. Endometrial cells derived from proliferative and secretory endometrial fragments, as well as menstrual endometrial fragments, are able to adhere to where the peritoneal mesothelium is damaged and the basement membrane or the interstitial ECM are exposed. Although an intact mesothelial lining may prevent adhesion and implantation of menstrual endometrial fragments, mesothelium can easily be damaged by surgery, inflammatory cells, and menstrual endometrium [139,140,141,142]. Some studies reported that isolated cells from menstrual endometrial fragments as well as non-cellular medium prepared from menstrual effluent are able to induce morphological alterations in the mesothelium, from an epithelial to a mesenchymal phenotype, and to damage it, creating its own adhesion sites at the mesothelial lining [143,144,145,146,147].

Adhesion of retrograde menstrual endometrium to the peritoneum is mediated by adhesion molecules that modulate cell-matrix and cell-cell attachments and are expressed by endometrial cells, including cadherins, integrins, proteoglycans such as syndecans, laminin-binding proteins, the immunoglobulin superfamily, and CD44. Integrins, syndecans, cadherins, CD44, and CD44’s binding partner hyaluronan have been studied extensively in endometrium and in endometriosis [138]. 

The integrins are transmembrane glycoproteins that modulate cell-matrix attachment and are involved in cell motility and invasion. The endometrial expression of integrins changes during the menstrual cycle under hormonal regulation. Altered patterns of expression that have been associated with endometriosis are able to express or to not express certain integrins independently of the hormones without cyclical modifications. Endometriosis is reported to have an overall highly variable and aberrant integrin expression as compared with eutopic endometrium. Fibronectin receptors were identified in glands of endometriosis implants but not in eutopic endometrial glands, suggesting that fibronectin receptors may contribute to the adhesion of endometriotic cells during menstruation [148,149,150,151,152].

Moreover, differences were observed between the eutopic endometrium of affected women and healthy women. The αvβ3 integrin is expressed in the endometrium at the time of implantation from 19-20 days of the menstrual cycle, and its absence suggests out of phase endometrium. In women with endometriosis, αvβ3 integrin is constantly absent in the eutopic endometrium although in the presence of in-phase histological features. This defect was associated with nulliparity and inversely related to the stage of disease [152,153]. 

Another family of ECM receptors is formed by the syndecans, four transmembrane-anchored proteoglycans that are expressed in the endometrium in a menstrual cycle-dependent manner [154]. Notably, the most ubiquitous member of the family, syndecan-4, was shown to be upregulated in the eutopic endometrium of endometriosis patients compared to an IVF control collective, and functional studies in an endometriotic cell line revealed that experimental syndecan-4 downregulation resulted in reduced invasiveness in vitro, and reduced expression of the cytoskeletal modulator Rac1, the transcription factor ATF-2, and MMP3 [155]. In addition, functional studies revealed a similar invasion-modulating role for the epithelial member of the syndecans family, syndecan-1: siRNA knockdown of this proteoglycan in endometriotic cells resulted in a substantial inhibition of Matrigel invasiveness, which was accompanied by a reduction of IL-6 secretion, MMP9 expression and MMP2 activity, and upregulation of plasminogen activator inhibitor-1 protein [124]. Overall, these data suggest that syndecans dysregulation in endometriosis contributes mechanistically to proteolytic remodeling, alterations in cell motility, and the inflammatory microenvironment, thus promoting invasive growth of endometriotic lesions.

Cadherins belong to a large family of transmembrane glycoproteins that mediate cell-cell adhesion and may suppress invasion inhibiting the escape of cells from their primary site. In vitro, tumor cells that express E-cadherin are retained by cell-cell adhesions, but when tumor cells do not express E-cadherin are no longer constrained and can invade. Therefore, E-cadherin is considered a central player in the development of cancer metastasis [156,157,158,159]. Immunohistochemical studies have reported that epithelial-glandular cells of menstrual effluent, eutopic endometrium, peritoneal fluid, peritoneum, and endometriosis express epithelial cadherin (E-cadherin), suggesting a role of E-cadherin in the maintenance of the endometrial epithelial architecture [151,160]. Endometriosis cells may share molecular mechanisms of invasion and metastasis with carcinoma cells that are related to the level of E-cadherin expression [156,157].

Finally, expression of the transmembrane adhesion molecule and stem cell marker CD44 is dysregulated in endometriosis [161], as exemplified by the correlation of high levels of soluble CD44 in the serum and peritoneal fluid of endometriosis patients with the severity of the disease. Indeed, preclinical data from animal models suggest that interference with CD44 function or with its binding partner, the ECM carbohydrate hyaluronan, may be a worthwhile therapeutic approach [162]. For example, inhibition of CD44 glycosylation was shown to decrease the attachment of endometrial cell lines to peritoneal mesothelial cells [163], and transplantation of endometrium from CD44-deficient mice into wild-type mice and vice versa resulting in a decreased formation of endometriotic lesions in vitro [164], suggesting an important role of CD44 in the adhesion of endometriotic cells to ectopic sites. Finally, pharmacological inhibition of biosynthesis of the CD44 substrate hyaluronan by 4-methylumbelliferone reduced angiogenesis in an in vivo mouse model of endometriosis [165], suggesting that interference with the CD44-hyaluronan axis may represent an approach that synchronously targets multiple molecular mechanisms of endometriosis.

### 3.6. Extracellular Matrix Remodeling and Matrix Metalloproteinases

The CAM models demonstrate that endometrium is able to adhere and subsequently degrade the ECM, suggesting that endometriotic lesion development requires ECM breakdown. The breakdown and remodeling of the ECM are mainly modulated by matrix metalloproteinases (MMPs) that degrade ECM components and are reported to be expressed by fragments of endometrium. MMPs are secreted in a latent preform requiring activation to acquire proteolytic activity and are inhibited by specific tissue inhibitors of MMPs (TIMPs). MMPs are structurally related but have different substrate specificity, cellular sources, and inducibility. Based on their substrate specificity, MMPs can be classified into collagenases, gelatinases, stromelysins, membrane-type MMPs, and other MMPs [166]. 

The activity of MMPs is modulated at the level of gene expression, at the level of latent proenzymes activation, and at the level of the inhibitory activity of TIMPs. Gene expression is modulated by hormones, growth factors, and inflammatory cytokines including IL-6, IL-1, epidermal growth factor, TNF-α, basic fibroblast growth factor (bFGF), and platelet-derived growth factor. Secreted latent proenzymes are activated by the stepwise activation of plasmin, which is considered the most potent activator in vivo, and by the activity of membrane-type MMPs, that are present at the cell surface and intracellularly. TIMPs are expressed by different types of cells and are present in the majority of tissues and body fluids [167,168,169]. 

MMPs are involved with highly regulated activity in many reproductive processes, including menstruation, ovulation, and embryo implantation [170,171]. The endometrial expression of MMPs is low during the proliferative phase, declines further during the early secretory phase and increases in the late secretory phase. Although MMPs activity in the endometrium is regulated by different hormones, cytokines, and growth factors, progesterone is a potent repressor both in vitro and in vivo. Progesterone might regulate MMP expression indirectly through the plasminogen activator pathway, increasing the levels of plasminogen activator inhibitor (PAI)-1 and thus reducing the plasmin-mediated activation of latent MMPs [172,173,174]. Moreover, locally produced retinoic acid and transforming growth factor-β (TGF-β) seem to be mediators of the progesterone suppression enhancing expression of TIMPs. Nevertheless, prematurely decreased progesterone levels not consistent with the peri-menstrual increased expression of MMPs and tissue degradation at focal points rather than throughout the different expression and regulation of MMPs entire endometrium are more likely than progesterone to be the primary modulator of endometrial collagenase activity [175,176,177].

After the initial attachment, the development of endometriotic lesion requires the invasion of adjacent tissues through the ECM degradation regulated by MMPs activity. This role of MMPs in the pathogenesis of endometriosis was proposed after finding ECM breakdown products in the peritoneal fluid of affected women [174]. Later, studies with artificial induced endometriosis, in mice and in the CAM, reported that endometriotic lesion development could be prevented by inhibiting the activity of MMPs [178,179]. Moreover, different expression and regulation of MMPs were demonstrated in women without and with endometriosis. This included an altered expression of specific MMPs and related TIMPs with an increased MMP/TIMP ratio in women affected by endometriosis as compared to healthy controls [63,166,180,181,182,183,184,185,186]. Specifically, in women with endometriosis, as compared to healthy controls, the expression of MMPs was reported enhanced in the secretory phase dominated by progesterone. In vitro studies reported an increased MMP-3 and MMP-7mRNA expression in the eutopic endometrium of women affected by endometriosis in the secretory phase. These results suggest that a contribution to the development of endometriosis comes from a defect in the response to the suppressing action of progesterone, a form of progesterone insensitivity [187].

Furthermore, evidence suggests that MMPs cleave not only ECM components but may also be implicated in the degradation of cytokines and growth factors, regulating tissue organization, angiogenesis, and cell survival. Therefore, MMP activity seems to be able to influence the initial lesion development as well as lesion survival and maintenance [188].

### 3.7. Angiogenesis

In CAM models, after 72 hours, a complete endometrium with glands and stroma was present in the CAM mesenchyme, and blood vessels were attracted from the CAM inside the endometrium fragment. These studies demonstrated that human endometrium is highly angiogenic and able to attract blood vessels from the surrounding tissue [189]. Angiogenesis is induced when vascular growth factors exceed inhibiting factors, and, although it is reported altered in pathological conditions such as cancer, chronic inflammation, and endometriosis, it is essential in a physiological process such as wound healing, growth, pregnancy and menstrual cycle [190,191]. Therefore, endometrial cells have physiologically angiogenic potential. In the eutopic endometrium during the menstrual cycle, angiogenesis is modulated by many factors, of which VEGF appears to be the most important for its ability to induce proliferation and migration of endothelial cell, vasodilation, and increased vascular permeability [190]. In the proliferative phase, estradiol induces VEGF-A production in endometrium resulting increased in the secretory phase and further increased prior to menstruation when endometrium becomes hypoxic as a result of vasoconstriction [192,193]. 

Increased endothelial cell proliferation, increased micro-vessel density, and higher levels of VEGF-A and angiopoietin-1 and -2mRNA expression were detected in the eutopic endometrium of patients with endometriosis than in the eutopic endometrium of disease-free women. These factors suggest a dysregulated angiogenic activity in the eutopic endometrium of women affected by endometriosis [64,191,194,195,196]. 

Focused studies on the vascularization of endometriotic lesions compared the vascular density and vessel diameter between white, black, and red lesions as well as between deep infiltrating lesions and endometriomas. No differences in the numbers of vessels were reported between different types of lesions, but red lesions have more vessels with a small diameter (<10 μm), whereas black lesions have more vessels with a larger diameter (>20 μm) [197]. The observations of endothelial cell proliferation with the development of blood vessels without smooth muscle in endometriotic lesions suggest that VEGF-A and angiogenesis are of significant value, particularly in early lesions. This was further suggested by studies investigating the soluble VEGF receptor sflt-1 that antagonizes VEGF-A action and was reported able to reduce the number of lesions formed in mice after intraperitoneal injection of endometrium [198]. More support to antiangiogenic therapy as effective in preventing the development of endometriosis has been provided by CAM model studies, in which anti-human VEGF factors administered to the CAM significantly decreased the vascular density of the CAM and prevented endometriosis-like lesion formation after the transplantation of human endometrium [199]. Moreover, after endometriotic lesions had been induced by transplanting human endometrium intraperitoneally, antiangiogenic agents resulted in a significant reduction of the number of endometriotic lesions in treated mice compared to control. The reduction in lesion number was related to a reduction of newly developed vessels, with mature vessels remaining unchanged [200,201]. Although VEGF is the key angiogenetic factor in endometriosis, further factors were reported involved in the angiogenesis of endometriotic lesions, including TGF-α, TGF-β, bFGF, angiopoietin, and hepatocyte growth factor [202,203,204]. Furthermore, recent results have indicated a role of the stem cell-related notch signaling pathway in sprouting angiogenesis of endometriotic lesions in an in vivo model [205].

### 3.8. Endometrial Stem Cells

More recently, endometrial stem progenitor cells were proposed as cells that give rise to the origin of endometriotic lesions, both ectopic or transplanted cells [206]. The human endometrium is subject to profound changes in tissue structure and function during the menstrual cycle, and the recovery of epithelial glands and stroma is produced by the endometrial progenitor stem cells within the basal layer, although some studies have suggested the origin may also be from bone marrow [206,207,208]. Endometrial stem cells demonstrated a high plastic capacity of differentiation by the characterization of several lines of cells with a different expression pattern of cell surface markers, endometrial localization, and clonal efficiency [209]. Recent evidence suggests the monoclonal origin of endometriotic cells within ovarian endometriomas, while peritoneal implants were reported to be polyclonal [210,211,212]. Moreover, as previously reported, the cells that give rise to endometriotic implants must undergo migration, adhesion, proliferation, and induction of angiogenesis. The endometrial stem cells have demonstrated the ability to activate all these characteristics [213]. As previously reported, it is proposed that physical and biochemical injuries caused by inflammatory cytokines and reactive oxygen species trigger the activation of endometrial stem cells inducing local production of estrogen and tissue injury-repair mechanisms such as cell cycle activation. On that basis, endometrial stem progenitor cells may be involved in the etiopathogenesis of benign and malignant endometrial aberrations such as endometriosis, endometrial hyperplasia, and endometrial cancer [214]. In general, endometrial stem progenitor cells have a long lifespan and trigger epigenetic mechanisms of protection from stress and senescence. They express genes such as Wnt/β-catenin, anti-apoptotic Bcl-2, cell cycle regulatory genes, and enzymes to repair DNA damage [206,209]. For all these characteristics, endometrial stem cells are supposed to be the cells that give rise to endometriotic implants instead of differentiated endometrial tissue fragments [7]. Of interest, endometrial stem progenitor cells can find their potential source from refluxed menstrual endometrium, Müllerian remnants from embryogenesis, as well as an hematogenic origin from the recruitment of circulating stem cell of bone marrow [215]. Notably, several stemness-associated molecular markers have been shown to be upregulated in the eutopic endometrium and ectopic lesions of endometriosis patients, including Msi1, SOX2, notch and numb [82,216,217], supporting the hypothesis of an involvement of stem cells in the pathogenetic process. Preclinical studies demonstrated that the microRNAs miR-145 and miR-200b were suitable tools to alter stemness-related properties of endometriotic cells, opening new therapeutic perspectives [218,219].

## 4. The Genetics of Endometriosis

The etiopathogenesis of endometriosis is still undefined and debated. To better understand the mechanism leading to the development of endometriosis as a disabling disease instead of spontaneous resolution of peritoneal implants, genetic studies offer an approach of paramount importance [3,220,221,222]. The evidence for a genetic contribution to the development of endometriosis comes from epidemiological studies that reported familial aggregation of the disease in humans [223,224,225,226,227] and primates [228]. Both hospital- and population-based studies reported higher rates of endometriosis among the relatives of affected women compared to healthy controls [224,225,229]. A study based on siblings, twins and familiars of affected woman reported a higher relative recurrence risk of 2.3 times as compared to the risk in the general population, although it is difficult to perform an accurate estimation because the prevalence in the general population is unknown and there is bias in the surgical diagnosis of endometriosis in siblings [226] and in the daughters of endometriosis patients (personal observation of D.C.M.). A genetic background for endometriosis was further supported by evidence that comes from larger studies in twins and in the population of Iceland [225,226,230,231,232]. Monozygotic twins show higher concordance for endometriosis than dizygotic twins [230,231], with intra-pair correlation rates of 0.52 versus 0.19 [232]. This data suggest that genetic factors influence about half of the risk for endometriosis development and estimate a heritability of 51% [232]. Further evidence comes from studies in the rhesus macaque animal model, that reported a familial aggregation of spontaneous endometriosis with a significantly higher coefficient of recurrence risk for full siblings (0.75) as compared to maternal (0.26) and paternal (0.18) half-siblings [228]. 

Although the familial aggregation of confounding risk factors, such as age at menarche, questioned the real role of genetic background in endometriosis risk [233], current evidence supports the genetic contribution to endometriosis development, and genetic approaches can be used to identify genes having a role in endometriosis risk, allowing a better definition of the etiopathogenetic pathways. Moreover, the identification of genetic markers may allow the development of more informative risk predictors as compared to family history, a better understanding of the pathogenesis providing new opportunities for drug discovery, and genetic profiles allow identification of co-morbidity, that may improve diagnosis and treatments.

### 4.1. Candidate Gene Studies

A general approach to investigate the role of genes in the etiopathogenesis of diseases is the study of specific candidate genes chosen based on the biological mechanisms known as contributors to the disease [234,235,236,237,238]. Nevertheless, for endometriosis, the definition of candidate genes is problematic because of the limited knowledge of etiopathogenetic mechanisms that may involve many genetic pathways and many genes. 

Studies have investigated genes involved in sex steroid pathways, detoxification pathways, cytokine signaling pathways, adhesion, and cell cycle regulation molecules and enzymes of the ECM [234,238]. Based on the estrogen-dependent nature of endometriosis, genes from pathways of sex hormones signaling and biosynthesis have been studied. These investigations provided limited evidence supporting the association between either PR or ER*α* and endometriosis [237], although a further large family-based study failed to demonstrate any association between endometriosis and PR [99]. Similarly, conflicting results were reported for the association between the gene expression of cytochrome P450 and endometriosis [239,240].

Detoxification pathways were investigated based on the supposed role of environmental estrogens in the etiopathogenesis of endometriosis, reporting questionable evidence [73,241]. Glutathione S-transferase enzymes are involved in the detoxification of different toxic compounds and carcinogens. Different studies investigated gene polymorphisms and reported evidence for an increased risk of endometriosis in the presence of specific enzyme variants. Nevertheless, significant heterogeneity between studies and publication bias suggests caution [235]. Similarly, a meta-analysis of studies that investigated the association between polymorphisms in the detoxification enzyme N-acetyltransferase 2 and endometriosis reported no association [236].

In general, many factors have contributed to the failure of candidate gene studies to provide new insights into the pathogenesis of endometriosis. A few of the reported associations have been investigated in an independent sample, as it is suggested before accepting the association with a disease, and the majority of replications failed to confirm previous results [238]. Study power is another cause of concerns both for initial and replication studies [242,243]. In complex diseases such as endometriosis, many genes, as well as environmental factors, may contribute to the etiopathogenesis; therefore, the effect size for the majority of common risk alleles is expected to be low, with odds ratios having a range of 1.1–1.5 [244,245,246]. On that basis, large sample sizes are required, and most candidate gene studies investigated small samples with inadequate power to detect the small contribution of all genes contributing to the risk of endometriosis [238,247,248]. Moreover, publication bias of significant compared to negative results [242,249], statistical and technical issues, and problems in experimental design provides further concerns [237]. In general, many gene variants with small effects can be considered associated with an increased risk of endometriosis [238].

### 4.2. Linkage and Association Studies

Linkage studies are performed in families with multiple cases and search for genomic regions shared more frequently than expected between relatives affected by the disease. These regions likely carry gene variants increasing the risk of developing the disease. These studies provided important results in disease related to single-gene mutations with Mendelian segregation [250,251,252]. Due to the high prevalence of endometriosis, the low recurrence in familiars, and the difficulties in ascertaining the healthy subjects, the best design was to analyze pairs of sisters both affected [252]. 

A genome analysis of DNA samples from 1176 sister-pair with both laparoscopically confirmed endometriosis and family members from Australian and UK was performed [253]. The study was designed with a power of 80% to detect a region with a recurrence risk to sisters of 1.35 [226]. The analysis identified significant linkage on chromosome 10 and on chromosome 20, although evidence for linkage was confirmed only in chromosome 10 for both Australian and UK families. A separate linkage study was conducted in families with three or more affected women and identified a significant linkage peak on chromosome 7p [254,255,256]. This suggests that in high-risk families, a locus with high penetrance for endometriosis risk may be located in this region.

Nevertheless, linkage studies are limited by the ability to identify a genomic region of interest but not a specific gene locus. Therefore, association studies are required to identify specific gene variants. Association studies, with or without family-based designs, compare gene variants frequency in women with endometriosis versus healthy controls. Because most human genome variants are single-base differences, methods to genotype large numbers of single nucleotide polymorphisms (SNPs) are now used for both linkage and association studies. Of interest, common SNPs (with a population frequency > 0.01) that are located close together are not independently inherited (linkage disequilibrium or LD) [257]. Therefore, a SNP can act as a marker for others, allowing the identification of the common variation in a particular region by typing a limited number of SNPs. With this technique, association studies investigated chromosome seven and 10 linkage regions identifying several genes implicated in endometriosis and endometrial cancer such as PTEN, homeobox protein EMX2, and the FGF receptor 2 gene (FGFR2). EMX2 is a transcription factor involved in the development of the reproductive tract and in the cyclicity of eutopic endometrium [258,259,260]. PTEN regulates proliferation and survival of cells and it is inactivated in the early events of endometrial hyperplasia development and ovarian and endometrial cancer pathogenesis [261]. FGFR2 has been associated with endometrial and breast cancer development [262,263]. Nevertheless, SNP studies, investigating common variants and variants related to endometrial or breast cancer of these three genes, suggested that the linkage signal is not related to these common variants, demonstrating no evidence for any association with the endometriosis [258,264]. However, a role for other unknown variants of these genes in the development of endometriosis cannot be completely excluded. Moreover, the hypothesis that different families may have different mutations of the same gene reduces the probability of identifying these specific variants. It is suggested that the results of linkage studies may be caused by multiple different variants of the same gene; the variant is the same across a single-family but is rare in the general population. Conversely, association studies require that investigated variants are common in the general population [258,264].

### 4.3. Genome-Wide Association Studies

The complex and multifactorial etiopathogenesis of endometriosis contributes to the failure of candidate gene, association, and linkage studies [245]. Genome-wide association (GWA) provided a new tool to identify genetic variants related to complex human diseases, with the use of genetic markers that include most of the common gene variants (up to 1 million SNPs) and can be screened in a single analysis [257].

Nevertheless, study design, particularly the definition of characteristics, traits of the disease being studied, and the choice of population controls, remains of paramount importance [265]. Moreover, cases and controls need to be well matched for ethnicity to reduce the false-positive rate, and standard, rigorous, and quality control procedures are required to reduce bias. Association results are reported as significant for every SNP that show a significant association as points above a stringent threshold determining the probability of finding a false positive. Once a genetic association is identified, replication provides an important safeguard against false-positive results and gives an independent and better estimate of the effect size [266]. The identified region can be subsequently examined to locate the genes relative to association signals and analyze the variation pattern because SNPs statistically associated with disease are probably not the causal variants, that usually are absent on the gene chip used for SNP detection, but correlate with the common variants genotyped on the chip. The causal variants probably lie near the regions of SNPs statistically associated with disease for the LD [267]. Of note, signals could identify both areas of the genome with genes and intragenic regions identifying regulatory regions of gene expression [244]. 

Well-powered endometriosis case-control GWA studies identified several genes as possible candidates in the pathogenesis of endometriosis. A Japanese GWA study identified a significant association with LD blocks of chromosome 1 near the cyclin-dependent kinase inhibitor 2B antisense RNA (CDKN2BAS) and the wingless-type MMTV integration site family 4 (WNT4) gene [268]. A smaller Japanese GWA study reported a significant association with LD blocks near IL-1 alpha proprotein. Notably, IL-1α has been shown to be elevated in both the peritoneal fluid and the serum of infertile women with endometriosis compared to healthy controls [269,270]. In a United States GWA study, SNP-association with endometriosis was reported with LD blocks near nuclear factor erythroid-derived 2-like 3 gene, typically expressed in placenta, and HOXA10 and HOXA11 genes, two candidate genes of the homeobox A transcription factors family. HOXA10 and HOXA11 have key roles in uterine embryogenesis and are expressed at high levels during the luteal phase. Of interest, studies reported that HOXA10 levels were not increased in women affected by endometriosis leading to infertility [271]. A meta-analysis incorporating available data about SNP-association reported different SNPs in the gene of interest between European descents and Japanese, likely reflecting the different genetic backgrounds of investigated populations. Nevertheless, WNT4 was confirmed as a candidate gene for endometriosis, with a signal that overlaps completely with an association signal for ovarian cancer, suggesting some common molecular pathways [220,222,271]. WNT4 is an interesting candidate involved in female reproductive tract embryogenesis, ovarian follicle development, and steroidogenesis. WNT4 was reported to be involved in numerous anomalies of the female genital tract. Moreover, the signaling pathway of WNT genes and WNT/β-catenin were reported to be associated in the control of different types of stem cells and in the resistance to apoptosis by the cell-cell interaction mediated by cadherins [268,272,273,274]. 

The International ENDOGENE Consortium, in addition to using SNP–disease associations, applied GWA data to investigate with statistical methods the percentage of disease risk variation attributable to genetic variants, and whether the disease status in an independent sample can be predicted by the disease status in another sample [226,275]. Analyses suggested that in moderate-to-severe endometriosis (Revised American Fertility Society (rAFS) classification stages III-IV) genetic load is higher than in minimal-to-mild disease (rAFS classification stages I-II) with strongest signals of SNP-association. This was subsequently confirmed by prediction analyses with SNP data from moderate-to-severe endometriosis of UK samples that predict moderate-to-severe disease in Australian samples better than data from all endometriosis cases. These results were confirmed by inverted analysis [226,275].

A recent meta-analysis incorporating available data from 11 GWA case-control studies, involving 17,045 endometriosis cases and 191,596 controls, identified five new loci significantly associated with endometriosis risk, that were reported involved in sex steroid hormones pathways. Overall, GWA case-control studies have identified 19 independent SNPs associated with endometriosis risk. Those include SNPs associated with endometriosis identified regions near the gene for ER*α*, regions upstream of the beta subunit of follicle-stimulating hormone (FSH), and the Growth Regulating Estrogen Receptor Binding 1 (GREB1), previously associated with breast cancer. Other regions identified genes involved in cell migration, adhesion, and proliferation, such as WNT4. Nevertheless, together, these identified SNPs are able to explain only 5.19% of the variance in endometriosis [220,222]. Therefore, the combined effects of all identified genes explain only a small fraction of the estimated 51% heritability of endometriosis [276]. This “missing” heritability may be explained, as reported for other complex diseases, by the small effect size of multiple single variants that contribute to disease risk and do not reach statistical significance. Additionally, SNPs on the current commercial chips may not tag well causal variants. Functional genetic variants that contribute to susceptibility for some common diseases, if rare, limit the performance of GWA studies that are designed to detect common variants and fail to identify low-frequency alleles [277,278,279,280].

## 5. The Epigenetics of Endometriosis

It has been demonstrated that a single lesion of endometriosis is monoclonal [210,211,212] and, based on gene expression profiling studies, a large number of genes are dysregulated in endometriosis [281,282,283,284,285,286,287]. Nevertheless, although the heritability of endometriosis is estimated to be 51% [232], it is difficult to identify specific genes consistently associated with endometriosis and with predictive power in identifying high-risk women [233,234]. During the development of endometriotic lesions from progenitor cells, it was proposed that the aberrations are acquired sequentially and usually without any change in the sequence of DNA (i.e., DNA mutations). Conversely, gene expression dysregulation in the cellular lineage is acquired and maintained by epigenetic processes in a heritable manner. Therefore, sequentially acquired and inherited changes at the level of gene transcription, post-transcriptional modulations, translation, and post-translational modifications are proposed as the common denominator explaining the hormonal, immunological, molecular, histological and cellular aberrations that characterize endometriosis. Furthermore, they may explain the SNPs-association with regulatory regions of gene expression instead of gene loci and the small effect of multiple single-gene variants in GWA studies [244]. At the same time, Bruner-Tran et al. [288] have investigated heritable epigenetic changes in mice in germ cells after exposure to 2,3,7,8-tetrachlorodibenzo-p-dioxin (TCDD) and demonstrated a transgenerational occurrence of several reproductive diseases that have been linked to endometriosis in women, although they could not determine if those changes lead to the development of endometriosis or were a consequence of the inflammatory nature of the disease. However, epigenetic markers occurring within the germline of mice are inheritable and can positively or negatively affect offspring [289,290]. These epigenetic changes potentially include a stable, heritable phenotype caused by chromosomal changes without DNA sequence alterations if they escape epigenetic reprogramming. If so, they are essential in tissue development and cell differentiation [291]. Moreover, epigenetic processes modulate phenomena such as genomic imprinting and X chromosome inactivation, and they are involved in aging and disease development [292,293,294]. Epigenetic processes involve dynamic changes in the chromatin structure influencing gene expression. Chromatin architecture is modulated by methylation of DNA (hypo- and hypermethylation correspond to gene expression and silencing, respectively), acetylation, ubiquitination, ADP-ribosylation and SUMOylation of histone proteins; and by non-histone proteins DNA-binding [295]. Moreover, epigenetic processes involve the expression of microRNAs (miRNAs). MiRNAs interact with mRNA, inhibiting translation, or inducing mRNA degradation [296,297,298,299]. In ectopic endometrial-like cells of endometriosis, the cellular identity and gene expression programs are defined and maintained by epigenetics that may have a key role in the pathogenesis. Of interest, these epigenetic aberrations are dynamic and reversible and may have potential implications for diagnosis, prognosis, and therapy of the disease. In summary, available evidence may suggest a role of epigenetics both in the development of endometriosis lesions and in the heritability of the disease.

### 5.1. Epigenetics in the Eutopic Endometrium

Epigenetic processes are involved in numerous mechanisms that modulate the gene expression in endometrial development during the menstrual cycle, resulting in coordinated functional and morphological changes [300]. The global methylation level of eutopic endometrium was reported higher in the proliferative phase as compared to the secretory phase of the menstrual cycle. This hypermethylation is consistent with the expression level of DNA methyltransferases 1 (DNMT1) that was reportedly higher in the proliferative and late secretory phase and lower in the mid-secretory phase. A similar expression level was reported for DNMT3A and DNMT3B, for which the gene expression levels were decreased by treatment with estrogen and progestin. Conversely, the expression level of DNMT1 was reported to be unchanged by hormones either due to technical limitations of the used method or because the estrogen and progestin act at the post-transcriptional level, reducing the stability of DNMT1 protein [301]. 

Similarly, histone modifications by acetylation seem to be involved in endometrial function, with histone acetylation levels reported as globally increased in the early proliferative phase and gradually reduced in the late proliferative phase until ovulation [300]. The histone deacetylase 1 (HDAC1), HDAC3, and two histone acetylases were reported to be constitutively expressed in the endometrium during the menstrual cycle, with HDAC1 having a reduced expression level in the secretory phase [302]. Of note, HDAC inhibitors (HDACIs) were able to determine a differentiation and morphological transformation in endometrium similar to the combined treatment with estrogen and progestin. These results suggest that histone modifications may play a role in the control of decidualization through the regulation of the function of ERs and PGE2-induced 17β-estradiol synthesis [4,303,304,305].

MiRNAs post-transcriptionally downregulate the expression of genes and seem to be involved in endometrium development during the menstrual cycle, similar to acetylation and methylation [306,307,308]. MiRNAs were identified in normal endometrium and in eutopic and ectopic endometrium of women affected by endometriosis [101]. An inverse correlation was reported between the expression level of specific miRNAs and the suppression of protein production derived from their target genes, such as aromatase and COX-2 [309]. Moreover, estrogen and progestins were reported able to modify the expression level of miRNAs in endometrial stromal and glandular epithelial cells. Progesterone may oppose estrogen action by fine-tuning gene expression modulating miRNAs, which seems to suppress genes involved in cycle progression and cell proliferation in the secretory-phase [309,310]. This involvement of miRNAs in endometrial cyclicity is consistent with data suggesting that miRNAs seem to have a role in embryo implantation and postnatal uterus development in the mouse [311,312].

### 5.2. Epigenetics in Endometriosis

Some forms of endometriosis could be considered an epigenetic disorder. A growing body of evidence suggests that epigenetics processes have a key role in the pathogenesis and pathophysiology of endometriosis [313]. Aberrant methylation, acetylation, post-translational modifications, and dysregulation of miRNAs expression were identified in eutopic as well as ectopic endometrium of affected woman and may have great potential as therapeutic targets or as biomarkers for diagnosis and recurrence risk prediction [314]. 

The first piece of evidence suggesting the role of epigenetic in the etiopathogenesis of endometriosis comes from studies reporting the hypermethylation of the HOXA10 gene promoter in eutopic endometrium of women with endometriosis as compared with healthy controls [315,316]. HOXA10 is a transcription factor belonging to the homeobox gene family and has a role in the development and function of the uterus. Eutopic endometrium expresses HOXA10 during the menstrual cycle with a high expression level in the mid-secretory phase. This high expression, corresponding to increased progesterone levels and to the time of implantation, suggests that HOXA10 may have a key role in establishing the conditions necessary for implantation [316]. Of interest, the hypermethylation of the HOXA10 gene promoter, which means gene silencing, is consistent with studies reporting that HOXA10 levels are reduced in the eutopic endometrium of women affected by endometriosis [271], which may be a cause of the impaired fertility of these women [316]. Animal models further confirmed the key role of HOXA10 gene silencing by promoter hypermethylation both in eutopic and ectopic endometrium [317]. Moreover, animal models suggest that aberrant methylation of the HOXA10 promoter can be induced by in utero exposure to endocrine-disrupting chemicals (EDC) such as diethylstilbestrol, with or without overexpression of DNMTs [318,319,320]. 

A growing body of evidence suggests that aberrant expression of interacting homeobox genes and WNT family genes, particularly the WNT/β-catenin pathway, may disrupt organogenesis of the urogenital tract, altering the cell differentiation/migration with ectopic implantation. The WNT/β-catenin signaling pathway is involved in stem cell function and maintenance. These pathways may support the Müllerian remnants hypothesis as well as may be implicated in the survival and implantation of transplanted endometrial stem progenitor cells [7]. Indeed, data suggest that estradiol supports disease progression by upregulating β-catenin, which is degraded when it does not bind E-cadherin. The inhibition of phosphorylation stabilizes β-catenin that reach the nucleus and interacts with transcription factors regulating proliferation and angiogenesis [61]. 

The epigenetic mechanism of methylation was identified as having a role in progesterone resistance of endometriosis. Studies demonstrated that the promoter of PR-B gene is hypermethylated in endometriosis and adenomyosis with subsequent reduced PR-B expression [321,322,323]. The relatively permanent nature of hypermethylation of PR-B promoter provides further explanation to the persistent PR-B downregulation and progesterone resistance in endometriosis. 

Further evidence confirmed the role of dysregulated methylation in the etiopathogenesis of endometriosis. Genes coding for three DNA methyltransferases, DNMT1, DNMT3A, and DNMT3B, were reported to be overexpressed in endometriosis. The aberrant expression of these enzymes involved in maintenance, as well as de novo methylation, suggests that hypermethylation in endometriosis may be widespread [324,325,326]. For example, in endometriotic cell lines lacking E-cadherin, whose deregulation was associated with endometriotic cells invasiveness, the gene was hypermethylated, suggesting that methylation of E-cadherin provides invasive properties [327,328]. Notably, E-cadherin expression is also regulated by miR-200b expression, suggesting that this important anti-invasive adhesion molecule is subject to epigenetic regulation [219]. Of interest, both hypoxia and inflammation were reported distinctly able to modulate DNMTs expression and can cause aberrant DNA methylation patterns [4,329]. 

**Figure 3:**
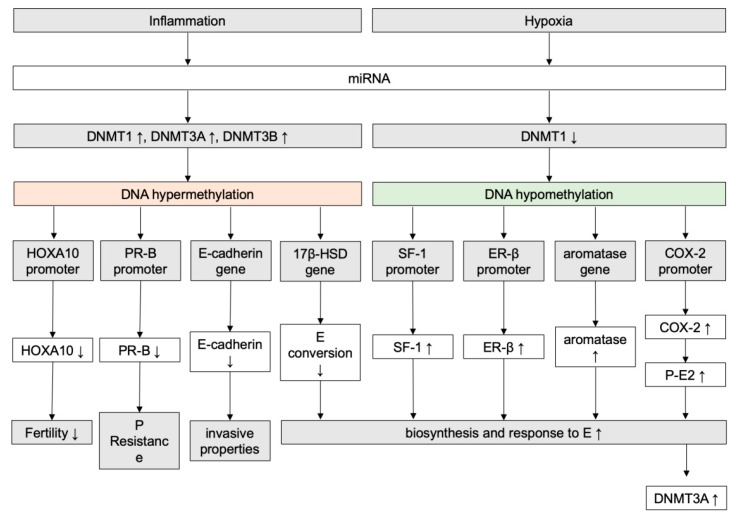
Epigenetic mechanisms of hypermethylation and hypomethylation involved in the etiopathogenesis of endometriosis. Hypoxia and inflammation regulate DNA methylation through microRNAs (miRNA), and genes coding for three DNA methyltransferases (DNMT) were reported overexpressed in endometriosis. microRNAs (miRNA); DNA methyltransferases (DNMT); Homeobox A transcription factor (HOXA10); Progesterone receptor B (PR-B); 17β-hydroxysteroid dehydrogenase (17β-HSD); Steroidogenic factor-1 (SF-1); Estrogen receptor β (ER-β); Cyclo-oxygenase type 2 (COX-2); Prostaglandin E2 (P-E2); Estrogen (E); Progesterone (P).

Endometriosis has both epigenetic mechanisms of hypermethylation as well as hypomethylation. The hypomethylation of specific genes and genes promoters that are hypermethylated in eutopic endometrium explains the altered expression in endometriosis of genes usually silenced in eutopic endometrium. Steroidogenic factor-1 (SF-1) is a transcriptional factor activating multiple genes for the biosynthesis of estrogen. SF-1 is not detected in stromal cells of eutopic endometrium because SF-1 promoter is usually hypermethylated. In endometriotic cells, the SF-1 promoter was reported to be hypomethylated, explaining the aberrant overexpression of SF-1 [330]. Similarly, the ER-β promoter and the aromatase gene were hypomethylated in endometriotic cells, while in eutopic endometrium, they are hypermethylated, providing an explanation as to why these genes are expressed in ectopic but not in normal endometrium. Consistently, the gene coding for 17β-HSD type 2 was reported hypermethylated. Moreover, the promoter of COX-2 was reported to be hypomethylated, explaining the higher expression of COX-2 and subsequent prostaglandin E2 synthesis, which in turn enhances 17β-estradiol production and elevate DNMT3A [4,314,331,332,333] (Figure 3).

Regarding the epigenetic regulation by histone acetylation, the balance between the HDACs and histone acetyltransferase activity regulates the gene transcription. Gene expression is promoted by the acetylation of lysine residue by the histone acetyltransferase, whereas is inhibited trough the removal of acetyl group by the HDACs [334]. Endometriotic lesions as well as eutopic endometrium of affected women were reported globally hypoacetylated as compared to the eutopic endometrium of controls, with consequent gene silencing [335]. This observation was consistent with the reported higher expression of HDAC1 and HDAC2 genes and lower levels of SIRT1 in the endometriotic lesions. Moreover, the lost modulation of HDAC expression by estrogen and progesterone was reported in ectopic implants [335,336,337].

Different genes are known to be involved in the etiopathogenesis of endometriosis by the methylation of their promoters and subsequent downregulation. The same genes and others were reported having the promoter hypoacetylated as well, such as the genes coding ER-*α*, HOXA10, E-cadherin, and different proapoptotic proteins involved in the cell cycle regulation [335,338]. 

The global aberrant acetylation is able to explain the altered expression of different genes both for downregulation by hypoacetylation and upregulation by hyperacetylation of the promoter, such as the higher level of SF-1 and hypoxia-inducible factor-1α [335,339].

Interestingly, recent evidence founds not only somatic inactivating mutations of the tumor suppressor ARID1A but also the loss of its expression in endometriotic foci [340]. ARID1A is known to encode the protein BAF250a, which participates in forming SWI/SNF chromatin remodeling complexes. Considering that this gene is frequently mutated in ovarian clear cell and endometrioid carcinomas as well as in uterine endometrioid carcinomas, the epigenetic loss of expression of BAF250a may underlie (at least in part) the potential degeneration of endometriotic tissue toward carcinogenesis. In particular, the loss of expression of the protein encoded by ARID1A (BAF250a) can dysregulate the suppression of cellular proliferation, which is normally modulated through a p53-dependent transcription fashion of several tumor suppressors, including CDKN1A (encoding p21) and SMAD3 [341]. Indeed, published data highlighted that tissue samples from patients who had undergone surgery for endometriosis-associated ovarian cancers or endometriotic ovarian cysts show loss of BAF250a expression in 22% endometrioid cancers, 47% of clear cell cases, 44% of contiguous endometriosis cases, and 8% of benign endometriotic ovarian cysts; in addition, the expression of phosphorylated AKT, γH2AX, BIM, and BAX was higher in endometriosis-associated ovarian cancers and contiguous endometriosis than in benign endometriosis, whereas expression of pATM, pCHK2, and Bcl2 was low [342]. Based on these pieces of evidence, the mutation of ARID1A and/or the loss of expression of BAF250a may appear as a promising strategy for molecular diagnosis of endometriosis-associated ovarian cancer [343].

The epigenetic post-transcriptional mRNA modulation by miRNAs expression has a potential role in the etiopathogenesis of endometriosis [4]. MiRNAs interacting with the correspondent mRNAs downregulate gene expression. Different studies report progressively different expression of many specific miRNAs from eutopic endometrium of healthy women, paired eutopic and ectopic endometrium of women with endometriosis, and ectopic endometrium. Most of these identified miRNAs target genes known to be differentially expressed in eutopic versus ectopic endometrium. Identified miRNA target genes includes those involved in hormone metabolism such as aromatase, PR, ER-α, and ER-β [310]; modulators of the inflammatory response such as COX-2, IL-6, IL-6 receptor, IL-8, and TGF-β; and the induction of apoptosis and angiogenesis such as Bcl-2, cyclin-D, and VEGF [4,136]. The inverse correlation between the expression level of miRNAs and that of target genes supports the hypothesis that altered expression of these specific miRNAs is involved in the pathogenesis of endometriosis [344]. Both upregulated and downregulated expression of specific miRNAs were reported in ectopic endometrium as compared with eutopic endometrium of women with endometriosis [345], as well as in eutopic or ectopic endometrium of women affected by endometriosis as compared with eutopic endometrium of healthy controls [346]. Functional studies in preclinical cell models of endometriosis have confirmed a mechanistic involvement of selected microRNAs in pathogenetically relevant processes, including modulation of cell proliferation by miR-10b and miR-145, of invasive growth by miR-10b, miR-200b, and miR-145, and of stem cell properties, as exemplified by miR-145 and miR-200b [124,218,219]. Of interest, although there is a discrepancy between studies in the identification of miRNAs having altered expression, no miRNAs were “misclassified.” These discrepancies can be explained by differences in study design and compared tissues. The comparison between eutopic and ectopic endometrium of affected women may not identify miRNAs associated with endometriosis that are aberrantly expressed in the same direction in both tissues. Finally, it should be noted that the role of miRNAs is more complex than a unidirectional negative regulation of gene expression. Evidence supports that many miRNAs are able to interact with transcription factors forming a network for gene regulation that yield negative as well as positive feedback loops [347]. Moreover, miRNAs have been reported to be both targets and regulators of other epigenetic mechanisms such as methylation and acetylation, and they resulted involved in hypoxia and inflammation signaling pathways [4,344].

### 5.3. Implications of Epigenetics in Diagnosis, Prognosis, and Therapy

The identification of epigenetic aberrations may provide promising tools for the diagnosis of endometriosis. DNA methylation markers, as well as other epigenetic aberrations that are present in the eutopic endometrium of women with endometriosis and absent in healthy controls, are present in the menstrual blood-derived from eutopic endometrium. The identification of these markers in the menstrual blood could provide high sensitivity and specificity in the identification of endometriosis with a minimally invasive approach [4,294]. These epigenetic markers may also provide prognostic information identifying patients at high risk of recurrence, allowing the tailoring of postoperative therapies and follow-up. For example, hypermethylation of the PR-B promoter identified in surgical tissue samples of endometriotic implants was related to a higher risk of recurrence [321,348].

The therapeutic implications are based on the reversible nature of epigenetic modifications. Therefore, enzymes involved in epigenetic mechanisms could be pharmacological targets. On that basis, HDACIs were investigated in vitro and in animal models as a potential therapy for endometriosis. In vitro, treatment with trichostatin A (TSA), an HDACI, elevated PR-B gene and protein expression, inhibited IL-1β-induced COX-2 expression, and inhibited cellular proliferation with cell cycle arrest in ectopic endometrial stromal cells, but not in eutopic cells [349,350,351]. In endometrial stromal cells, TSA upregulated Peroxisome proliferator-activated receptor (PPAR)γ expression which inhibits VEGF expression, angiogenesis, and TNF-induced IL-8 production [352,353,354]. Moreover, TSA attenuated invasion inducing E-cadherin expression [327]. In animal models with induced endometriosis, TSA represses endometriosis, reducing the size of ectopic implants as compared with no-treatment [59,355]. Treatments with other HDACIs and demethylation agents in both in vitro and in vivo studies provided similar promising results [59,323,356].

Data about the use of HDACI in humans derives from the use of Valproic Acid (VPA), an HDACI with known pharmacology. In women with adenomyosis, VPA reduces the amount of menses and dysmenorrhea. This could be explained by the fact that HDACIs suppress the expression of TNF-α-induced tissue factor and VEGF receptor as reported in both in vitro and in vivo studies. Both these pathways are involved in abnormal uterine bleeding and resulted in overexpressed in endometriosis [357,358,359,360]. VPA tested on patients was well tolerated, and after two months had reduced pain symptoms, reduced amount of menses, and reduced uterine size [361,362,363]. This evidence suggests that HDACIs have a potential role as therapy in endometriosis and/or adenomyosis [364].

## 6. Conclusions

The etiopathogenesis of endometriosis is a multifactorial process that leads to the development of an extremely heterogeneous disease characterized by the variable acquisition and loss of cellular functions. Its origin would appear to be from Müllerian or non-Müllerian stem cells with endometrial differentiation that can potentially originate from stem cells of the endometrial basal layer, present in Müllerian remnants, in the blood originating from bone marrow, or from the peritoneum. These stem cells have the ability of the endometrium to regenerate cyclically by mechanisms of tissue regeneration and angiogenesis in response to hypoxia, which seem to play a key role when they are dysregulated in the development of endometriosis. What determines the presence of such cells in the peritoneal cavity can occur during the development of the embryos as well as during each menstrual cycle, and what leads to the development of endometriosis is a complex process in which play a large number of interconnected factors potentially both inherited and acquired. Genetic studies have confirmed that endometriosis has a genetic nature, but at the same time, this predisposition is complex. It is constituted by the combined action of several genes with limited influence. At the same time, the epigenetic mechanisms underlying endometriosis control many of the processes of acquisition and maintenance of immunologic, immunohistochemical, histological and biological aberrations that characterize both the eutopic and ectopic endometrium in patients affected by endometriosis. However, what triggers such epigenetic alterations is not clear and may be both genetically and epigenetically inherited, or it may be acquired by the particular combination of several factors linked to the persistent presence of menstrual reflux in the peritoneal cavity as well as exogenous factors playing a critical role. Once started, the process is variable and can lead to the development of endometriosis through the progressive acquisition of alterations to the physiological processes of the endometrium, including the altered hormonal physiology, and modulating the interaction between endometriosis and the inflammatory response by subjugating it. However, the heterogeneity of endometriosis and the different contexts in which it develops suggests that a single etiopathogenetic explaining model is not sufficient.

## Abbreviations

MeSH	Medical Subject Headings
CAM	Chorioallantoic membrane
17β-HSD	17β-hydroxysteroid dehydrogenase
COX-2	Cyclo-oxygenase type 2
TIAR	Tissue injury and repair
TNF	Tumor necrosis factor
IL	Interleukin
VEGF	Vascular endothelial growth factor
Bcl-2	Antiapoptotic protein B cell lymphoma 2
NK cells	Natural Killer cells
sICAM-1	Soluble form of intercellular adhesion molecule-1
LFA-1	Leukocyte function antigen-1
MAPK	Mitogen-activated protein kinase
ECM	Extracellular matrix
HLA-1	Human leukocyte antigen class I
INF	Interferon
TGF-β	Transforming growth factor-β
E-cadherin	Epithelial cadherin
MMPs	Matrix metalloproteinases
TIMPs	Tissue inhibitors of MMPs
bFGF	Basic fibroblast growth factor
PAI	Plasminogen activator inhibitor
PR	Progesterone receptor
ER	Estrogen receptor
SNPs	Single nucleotide polymorphisms
LD	Linkage disequilibrium
FGF	Fibroblast growth factor
FGFR2	FGF receptor 2 gene
GWA	Genome-wide association
CDKN2BAS	Cyclin-dependent kinase inhibitor 2B antisense RNA
MMTV	Mouse mammary tumor virus
WNT4	Wingless-type mouse mammary tumor virus integration site family 4
rAFS	Revised American Fertility Society
FSH	Follicle-stimulating hormone
GREB1	Growth Regulating Estrogen Receptor Binding 1
TCDD	2,3,7,8-tetrachlorodibenzo-p-dioxin
miRNAs	microRNAs
DNMT	DNA methyltransferase
HDAC	Histone deacetylase
HDACIs	Histone deacetylase inhibitors
EDC	Endocrine-disrupting chemicals
SF-1	Steroidogenic factor-1
HOXA10	Homeobox A transcription factor
P-E2	Prostaglandin E2
E	Estrogen
P	Progesterone
TSA	Trichostatin A
PPAR	Peroxisome proliferator-activated receptor
VPA	Valproic acid
